# P02-013 - TH17 cells and regulatory T cells in TRAPS

**DOI:** 10.1186/1546-0096-11-S1-A120

**Published:** 2013-11-08

**Authors:** PM Vaitla, E Drewe, EM McDermott, I Todd, L Fairclough

**Affiliations:** 1 Immunology, Nottingham University Hospitals NHS Trust, Nottingham, UK; 2 Immunology, University of Nottingham, Nottingham, UK

## Introduction

The immunopathogenesis of TRAPS is thought to centre on activation of the innate immune system resulting in episodic inflammation. The adaptive immune system, Tregs and Th17 T cells, has not been studied in TRAPS. Different anti-TNF agents have different clinical effects on TRAPS i.e. etanercept has benefits but adalimumab and infliximab may trigger flares of TRAPS. It has been shown that different anti-TNF agents have differential effects on regulatory T cells in rheumatoid arthritis i.e. adalimumab but not etanercept induces peripheral Tregs which have the ability to suppress Th17 cells. We considered whether Tregs and Th17 cells in TRAPS could underpin differential response to biologicals.

## Objectives

To investigate whether there are differences in the numbers of regulatory T cells and Th17 cells in TRAPS patients compared to controls.

## Methods

Regulatory T cells from 5 patients with C33Y mutation TRAPS and 5 healthy controls were analysed by flow cytometric analysis on fresh blood. Lymphocytes which were CD4+/CD25 high were regarded as regulatory T cells. Th17 cells were also analysed by flow cytometric method involving cytoplasmic staining for IL17. Cells which were CD3+/CD8-/IL17+ were regarded as Th17 cells.

## Results

The TRAPS patients included 2 patients on anakinra, 1 on etanercept, 1 on tocilizumab and 1 on canakinumab. The mean number of CD4+/CD25high cells was 21.4 cells/µl in TRAPS patients compared to 13.2 cells/ µl in controls. Although TRAPS patients had higher numbers of CD25 high T cells, this did not reach statistical significance with a P value of 0.107. The mean percentage of Th17 cells in TRAPS patients was 1.92% compared to 1.72% in controls; p value of 0.649. Due to small numbers, it was not possible to comment on any differences between different biological therapies.

## Conclusion

Although CD4+/CD25high regulatory T cells appeared to be higher in the TRAPS patients, this did not reach statistical significance which could reflect small numbers studied. There were no significant differences in Th17 cells between the 2 groups. We suggest a further study of a larger group of patients using intracellular FoxP3 staining to further investigate the increased regulatory T cells apparent in TRAPS.

## Disclosure of interest

None declared.

